# Strategies to improve recruitment to multicomponent group programs for overweight and obesity: a systematic review

**DOI:** 10.3389/frhs.2025.1404181

**Published:** 2025-05-29

**Authors:** Inanna Reinsperger, Sarah Wolf, Ingrid Zechmeister-Koss

**Affiliations:** HTA Austria—Austrian Institute for Health Technology Assessment GmbH, Vienna, Austria

**Keywords:** obesity, overweight, group program, recruitment, barriers, participation

## Abstract

**Objective:**

Multicomponent programs are recommended for the treatment of children, adolescents, and adults with overweight or obesity. However, program providers often face difficulties reaching their target groups. This systematic review aimed at identifying recruitment strategies for multicomponent overweight and obesity programs in group settings and at summarizing barriers and facilitators for participation.

**Methods:**

We searched electronic databases (MEDLINE, CINAHL, The Cochrane Library, PsycInfo, Web of Science) and included primary studies reporting on recruitment strategies for multicomponent group programs for children, adolescents, and adults with overweight or obesity. All study designs were eligible for inclusion. Study characteristics, recruitment strategies as well as barriers and facilitators were extracted from the included articles, summarized in a table format, and synthesized narratively.

**Results:**

Of the 1,082 articles identified through the systematic literature search, 16 studies met the inclusion criteria and were included in the analysis. Eleven focused on children and adolescents, and five on adults. Recruitment strategies were categorized into active (e.g., referral by health professionals, direct contact) and passive methods (e.g., media advertising, flyers, posters). In most studies, a combination of several active and passive methods was applied or recommended. For socioeconomically disadvantaged groups, some targeted strategies were identified, e.g., recruitment in specific locations or through trained peers. Several possible barriers to recruitment were mentioned in the included studies, e.g., stigmatization, lack of time and resources of the healthcare staff, organizational barriers, lack of motivation of potential participants.

**Conclusion:**

This systematic review identified several active and passive strategies for recruiting children, adolescents, and adults with overweight and obesity into multicomponent group programs. The overview of possible recruitment methods is a valuable decision support to be used by program providers when designing new or adapting existing programs.

## 1 Introduction

The World Health Organization (WHO) describes obesity in children, adolescents, and adults as a serious global challenge for public health in the 21st century. It is a significant determinant of disability and death in the WHO European Region, where almost 60% of adults, one in four adolescents and one in three school-aged children live with overweight or obesity (1). Several medical conditions are associated with obesity, including cancer, cardiovascular diseases, diabetes, musculoskeletal complications, and mental health problems (1). Overweight in children and adolescents not only affects their immediate health but is associated with a higher risk and earlier onset of various non-communicable diseases such as cardiovascular disease and type 2 diabetes. It can also have adverse psychosocial consequences, such as discrimination and bullying (2).

Multicomponent behavioral change interventions, offered as one-to-one sessions or in a group, are the standard treatment of overweight and obesity (3, 4). These interventions include diet, physical activity, and behavior change elements (1). Drug therapy can be considered as a complement to lifestyle therapies if the body mass index (BMI) is above 30 kg/m^2^ or the patient has comorbidities. Bariatric surgery can be an option for adults with a BMI above 40 kg/m^2^ or between 35 and 40 kg/m^2^ with comorbidities (4). For children and adolescents, such multicomponent behavioral change interventions should be family-oriented and multidisciplinary (3).

Often, the providers of such (group) programs face difficulties reaching their target groups and motivating them to participate. These recruiting difficulties have been described for several population groups, e.g., children and their families (5), and young adults (6). Socioeconomically disadvantaged and ethnic minority groups have a higher risk for obesity and the associated morbidity and mortality, but may experience various barriers in accessing specific obesity management health services (1, 7).

Therefore, this article aimed to systematically review the scientific literature on strategies for recruiting children, adolescents, and adults with overweight or obesity and motivating them to take part in multicomponent group programs for overweight and obesity, including nutrition, exercise, and behavioral therapy elements. A particular focus was on reaching socioeconomically disadvantaged people. The information on successful recruitment strategies can be used to develop new or adapt existing programs so that they can reach their target groups more effectively.

## 2 Materials and methods

This systematic review was conducted as part of a larger project on multicomponent group programs for children, adolescents, and adults with overweight or obesity. The project protocol was published on the Open Science Framework (OSF) website (8). The systematic review was conducted following most of the items of the PRISMA 2020 Checklist for systematic reviews (9).

### 2.1 Systematic literature search

The following electronic databases were searched from May 24 to May 26, 2023: MEDLINE, CINAHL, The Cochrane Library, PsycInfo and Web of Science. An information specialist conducted the database search. We also manually searched the reference lists of eligible articles for further relevant articles. The search strategies for each database are included in the Supplementary Material.

### 2.2 Inclusion and exclusion criteria

The literature was selected according to the pre-defined inclusion and exclusion criteria (see Table 1). We included studies on programs for children and adolescents from four to 18 years and adults from 18 to 65 years with overweight or grade 1 obesity. Studies on interventions for children under the age of four years and adults older than 65 years were excluded, as were interventions for people with more severe obesity. We included primary studies of all study designs (e.g., RCTs, qualitative studies) that focused on the accessibility of the respective target groups and provided information regarding the recruitment strategies and/or the barriers and facilitators for recruitment. The recruitment strategies and outcomes were described for multicomponent overweight/obesity group programs, including nutrition, exercise, and behavioral therapy elements. We excluded studies of other programs, e.g., focusing on only one aspect, such as nutrition, or with individual rather than group settings. Studies that investigated general facilitators and barriers without being linked to a specific overweight/obesity program were also excluded, as were studies on specific programs for women who are pregnant or have recently given birth.

**Table 1:** Inclusion and exclusion criteria (PICO).

Component	Inclusion criteria	Exclusion criteria
Population	– Children & adolescents (4–18 yrs) with overweight or obesity (according to the respective national classification*) – Adults (18–65 yrs) with overweight (BMI >25–30) or grade 1 obesity (>30–34.9) (WHO classification) Specific focus: people with a BMI in the overweight range and socially disadvantaged groups	– Children & adolescents with extreme obesity (according to the respective national classification), children <4 yrs – Adults with degree >1 obesity (BMI >34.9; WHO classification), adults >65 yrs – Pregnant and postpartum women
Intervention	– General strategies/methods for effective recruitment of the target groups – Specific strategies/methods for generational groups/people with a BMI in the overweight range/socially disadvantaged people to multicomponent group programs, including nutrition, exercise, and behavioral therapy elements	– Recruitment strategies/methods to other programs, e.g., focusing on nutritional therapy or exercise alone, individual settings, inpatient programs, school-based or workplace-related programs…
Comparison	Control interventions reported in the studies (if available, depending on the study design)	–
Outcomes	– Characteristics of the recruitment strategies and methods – Quantitative and/or qualitative data on: ○ Recruitment (e.g., recruitment rate) ○ Participation (e.g., participation rate) – Barriers and facilitators for recruitment	–
Study designs	Primary studies (no restriction of the study design)	Systematic reviews
Language	German, English	All other languages

BMI, body mass index; WHO, world health organization; yrs, years.

* e.g., Germany/Austria: overweight = BMI >90th percentile, obesity = BMI >97th percentile (3); United States: overweight = BMI between the 85th and the 95th percentile, obesity = BMI >95th percentile (4).

### 2.3 Literature selection process

In the first step, titles and abstracts of the identified articles were screened using the website Rayyan (https://www.rayyan.ai/). Articles that appeared relevant were assessed in full text. The abstract and full-text screening was primarily conducted by one author (IR). Uncertainties regarding the study selection were resolved by discussion and consensus with the co-author (SW) or involving a third person (IZK).

### 2.4 Data extraction and synthesis

The data were extracted from the included articles and put into pre-defined tables for children/adolescents and adults. We extracted the following information:

– Study characteristics: author, year, country, study design, aim, methods of data collection and analysis
– Target population, description of the intervention/program (setting, content, duration, number of sessions, team), control intervention
– Recruitment setting and recruiting person
– Recruitment strategies: Categorization into passive and active recruitment strategies: passive recruitment strategies are those where potential participants must initiate contact with researchers after being exposed to recruitment materials, such as flyers, posters, newspaper advertisements, or social media posts. These strategies rely on individuals identifying themselves as eligible and interested. In contrast, active recruitment strategies involve direct outreach by researchers or program staff to identify and engage potential participants, including direct physician referrals, targeted mailings to eligible individuals, telephone calls, face-to-face recruitment in clinics or community settings, and community outreach events. The distinguishing feature between these approaches is who initiates contact—in passive recruitment, potential participants make first contact, while in active recruitment, the research team initiates the engagement (10).
– General recommendations to improve recruitment
– Recruitment rates
– Barriers and facilitators for participation

The program descriptions in the articles that focused primarily on recruitment strategies were sometimes incomplete. In such cases, we additionally searched for related publications and extracted the information on the intervention (e.g., content, duration, setting) from these articles.

We analyzed the data narratively (separately for different age groups/generations, where reported). The data extraction and synthesis were performed by one author (IR) and reviewed by another author (SW). The complete data extraction tables are available on request from the corresponding author.

### 2.5 Quality assessment

The critical appraisal of the methodological quality of the included studies was carried out using the Quality Assessment with Diverse Studies (QuADS) checklist (11) by one author (SW) and reviewed by another author (IR). The QuADS checklist is an assessment based on 13 criteria that can be used for quantitative, qualitative, and mixed methods studies. The assessment of study quality does not result in a final score.

## 3 Results

### 3.1 Characteristics of the included studies

**Figure 1:**
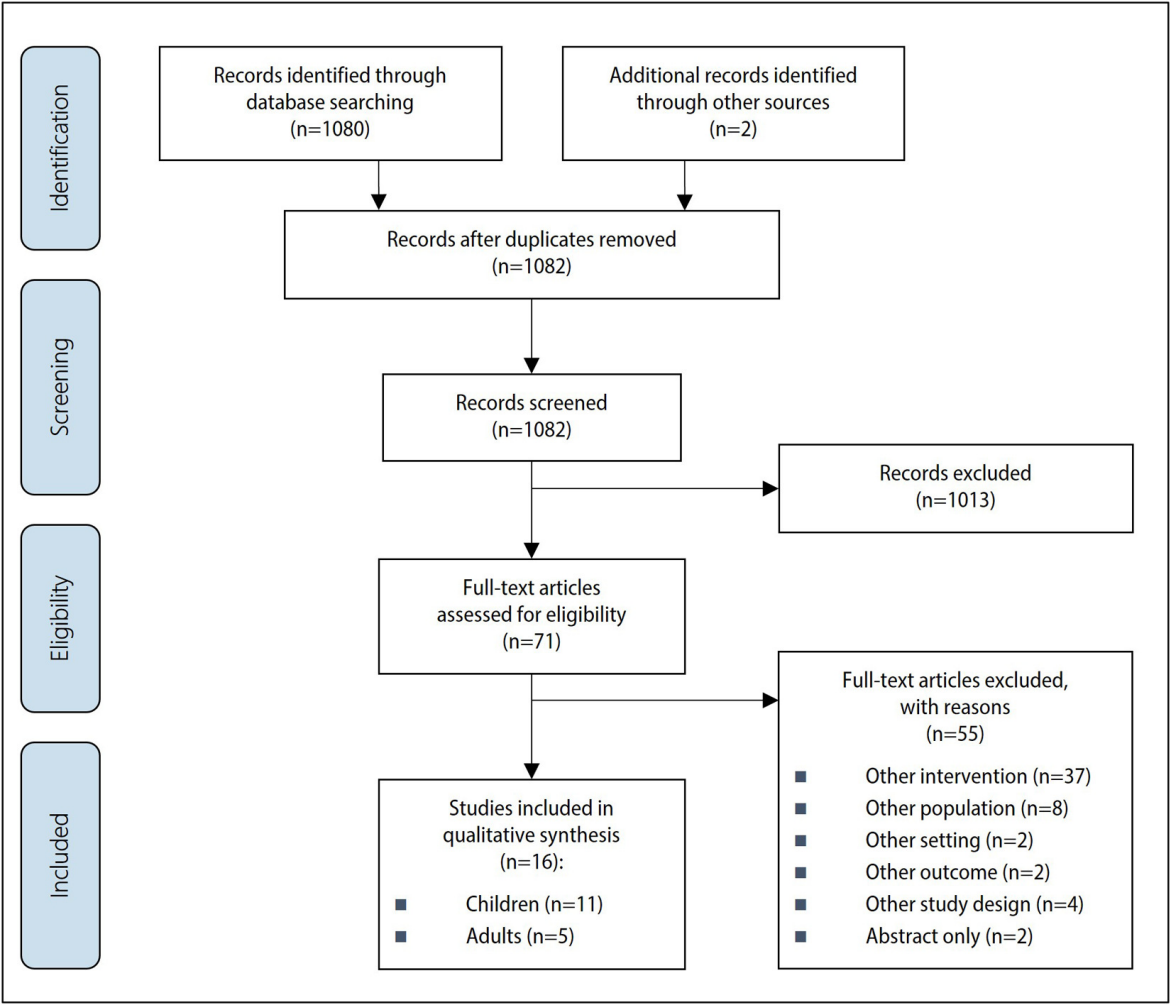
PRISMA flow chart.

The systematic literature search and additional hand search yielded a total of 1,082 sources that were available for the literature selection. Based on the pre-defined inclusion criteria, we excluded 1,013 sources after the abstract screening and reviewed 71 full texts for inclusion. Sixteen studies met the inclusion criteria and were included in the analysis (see Figure 1). Eleven of the 16 studies focused on children and adolescents (12–22) and five on adults (10, 23–26). The articles were published between 2008 (21) and 2022 (14). Ten of the 16 studies were conducted in the United States (10, 12–14, 18, 19, 21, 23–25), two in the United Kingdom (16, 26), two in Australia (20, 22) and one each in the Netherlands (17) and in Germany (15). The objectives of the included studies mainly were to describe, evaluate and compare the different recruitment strategies (10, 12–16, 18–21, 23, 24, 26). Two studies focused primarily on the identification and description of barriers and facilitating factors for the recruitment of the respective target groups (17, 22) and another study aimed to identify appropriate recruitment methods with the help of qualitative research (group discussions) (25).

Regarding the study design, half of the included studies were evaluations or descriptions of different recruitment strategies for RCTs (10, 12, 15, 16, 18, 20, 24, 26), whereas another study analyzed the recruitment to a program (21). Two further studies were RCTs that compared different recruitment strategies (14, 23). Another study conducted a secondary analysis of data from three clinical trials (19). Four studies described the results of qualitative surveys (interviews, focus groups) (13, 17, 22, 25).

Concerning the type of data collection and analysis, six studies reported results from qualitative data collection, either exclusively (13, 17, 22, 25) or as a supplement to quantitative data (16, 24). The qualitative data were collected in interviews (13, 16, 17, 22), focus groups/group discussions (22, 24, 25) or as written notes by the recruiters (24), and mostly related to the identification of barriers (13, 17, 22, 24). Quantitative methods were used in twelve studies, but most studies did not describe these in detail. Purely descriptive methods can be found in seven studies (10, 15, 16, 20, 21, 24, 26), mostly involving a quantitative description of the participants depending on the recruitment strategy or the response rates. In addition to descriptive methods, three studies (12, 18, 19) also used correlation methods for secondary analyses of data from other studies, e.g., to determine the relationship between the number of people recruited and specific recruitment methods (18, 19) or characteristics (e.g., BMI, age) (12). The two RCTs (14, 23) tested the effect of different recruitment strategies. The barriers and facilitators were generally described narratively, mainly in the discussion of the publications, and how these barriers and facilitators were collected was not specified. An exception was the qualitative studies, which primarily focused on identifying barriers (13, 17, 22).

The study characteristics are summarized in Table 2.

**Table 2:** Summary of study characteristics.

Author, year (reference)	Country	Target population characteristics	Program description, setting, program duration	Study design	Methods of data collection and synthesis
Children and adolescents					
Barlow et al., (12)	US	Children aged 2–12 yrs with overweight or obesity from racially/ethnically diverse, low-income areas	TX CORD study: RCT that compared a community-based program, which used *Mind, Exercise, Nutrition… Do it!* (MEND) and an adapted *Coordinated Approach to Child Health* (MEND-CATCH) program, with a healthcare-based program YMCA facility; 12 months	Evaluation of recruitment to an RCT	Quantitative (comparison of demographic and anthropometric characteristics, various statistical tests to evaluate for differences, e.g., between sites, by BMI status, within age groups)
Brock et al., (13)	US	Families of a child with a BMI ≥85th percentile, English-speaking	Comparison of 2 programs: – *iChoose*: high-intensity family-based program with family sessions, telephone support calls, exercise, workbooks – *Family Connections*: low-intensity program Setting NR; 3 months	Qualitative study (interviews with academic and community partners and parent study participants)	Qualitative (interviews, using a capacity framework guided by community-based participatory research principles, content analysis, and thematic categorization)
Darden et al., (14)	US	Children aged 6–11 yrs with overweight or obesity and their parents or caregivers, English speaking, rural residence	*iAmHealthy* Behavioral Intervention: family-based behavioral group intervention delivered via interactive televideo to caregivers and children from rural areas Online setting; 6 months	Cluster-RCT	Quantitative (statistical testing of recruitment methods to assess whether either or both of the recruitment methods were appropriate to fill a full-scale trial)
Finne et al., (15)	Germany	Children aged 8–16 yrs with overweight (not obesity) and their parents	*Obeldicks LIGHT*: outpatient training, based on the intervention Obeldicks for obese children and adolescents Clinic; 6 months	Evaluation of recruitment strategies to an RCT	Quantitative (interview/ questionnaire on recruitment source; descriptive statistics and narrative description of recruitment sources)
Fleming et al., (16)	UK	Children aged 6–11 yrs with overweight or obesity	*Families for Health* study: RCT evaluating the effectiveness of a family-based community program with parallel groups for parents/carers and children Community venue; 10 weeks	Evaluation of recruitment methods to an RCT (including qualitative data from interviews with parents/carers)	Quantitative and qualitative (interview of potential participants on recruitment source; descriptive statistics and narrative description of recruitment methods and outcomes, semi-structured interviews with parents/carers)
Gerards et al., (17)	Netherlands	Parents of children aged 4 yrs with overweight	Parent-focused group program aimed at the prevention of excessive weight gain in 4-year-old overweight children Setting NR; 14 weeks	Qualitative theory-based study applying semi-structured interviews	Qualitative (interviews with professionals on recruitment barriers, categorization of barriers into 5 categories based in the implementation theory by Fleuren et al.)
Huffman et al., (18)	US	African American families with an adolescent aged 11–16 yrs with overweight or obesity	*Families Improving Together* (FIT): RCT to test the efficacy of integrating cultural tailoring, positive parenting and motivational strategies into a comprehensive curriculum for weight loss in African American adolescents Setting NR; 8 weeks	Secondary analysis of recruitment data available for an RCT	Quantitative (secondary analysis of recruitment data: coding of recruitment strategies, descriptive statistics, logistic regression)
McCullough et al., (19)	US	School-age opt-in group: children aged 8–12 yrs, BMI ≥85th percentile, living in rural communities Preschool opt-in group: children aged 3–7 yrs, BMI ≥85th percentile, living in rural communities Preschool opt-out group: children aged 2–5 yrs, BMI ≥95th percentile	3 family-based interventions: – *Extension Family Lifestyle intervention project for Kids* (E-FLIP for Kids) – *Community Health Lifestyle Intervention for Rural Preschoolers* (CHIRP) – *Learning about Activity and Understanding Nutrition for Child Health* (LAUNCH) Community, clinic, at home; 4 to 12 months	Secondary data analysis from 3 weight management trials	Quantitative (secondary analysis of data from 3 trials: recruitment rate calculations, chi-square analyses to compare different samples)
Nguyen et al., (20)	Australia	Adolescents aged 13–16 yrs with overweight or obesity	*Loozit®*: RCT aimed to evaluate the effect of additional therapeutic contact via telephone coaching and electronic communication as an adjunct to the Loozit community-based weight management Community health center; 2 years	Evaluation of recruitment strategies to an RCT	Quantitative (descriptive statistics and narrative description of recruitment strategies)
Rice et al., (21)	US	Children aged 7–17 yrs with a BMI >85th percentile	Facility-based program with individual and group sessions for the children and parents Private health club; 12 months	Evaluation of recruitment strategies to a youth weight-management programme	Quantitative (descriptive statistics and narrative description of recruitment strategies)
Smith et al., (22)	Australia	Adolescents aged 12–16 yrs with a BMI >85th percentile	*Curtin University Activity, Food and Attitudes Programme* (CAFAP): healthy lifestyle program for adolescents and their parents Setting NR; 8 weeks	Qualitative study (focus groups and semi-structured interviews with adolescents, parents and community stakeholders)	Qualitative (focus groups and interviews on barriers and enablers; transcription and thematic analysis using inductive techniques)
Adults					
Befort et al., (10)	US	Adults aged 20 to 75 yrs with a BMI of 30 to 45, living in a rural location	Comparison of group visits conducted after hours within the local practice that emphasize coordinated, comprehensive care with enhanced access or group conference call visits Primary care practice; 24 months	Evaluation of recruitment strategies to a cluster-RCT	Quantitative (narrative description of recruitment sources and response rates)
Brown et al., (23)	US	Women aged 21 to 75 yrs from different racial/ ethnical backgrounds	*Stability Skills First*: 20-week behavioural weight-loss program and 8-week problem-solving skills maintenance module Research center; 6 months followed by a 12-month follow-up period	RCT	Quantitative (testing of the effect of two direct mail characteristics)
Chang et al., (24)	US	Non-pregnant African American and white women aged 18 to 34 yrs, BMI between 25 and 39.9, having a youngest child aged between 6 weeks and 3.5 yrs	*Mothers In Motion* (MIM): pilot intervention with interactive DVD plus a series of peer support group teleconferences Online setting; 10 weeks	Description of recruitment strategies to an RCT and qualitative evaluation (recruiter's notes, focus groups)	Quantitative and qualitative (survey data collected by telephone interviews, evaluation of procedures via recruiters’ log notes and focus groups)
Corsino et al., (25)	US	Young adults aged 18 to 35 yrs with overweight or obesity (BMI ≥25)	*Cellphone Intervention for You* (CITY): behavioral intervention delivered via a smartphone app or through personal coaching enhanced by self-monitoring via smartphone Setting NR; 24 months	Qualitative study conducted during the formative phase of an RCT	Qualitative [group discussions with members of the target population to inform development of recruitment strategies, using the nominal group technique (NGT); grouping of themes, ranking by the participants]
Randell et al., (26)	UK	Adults aged 18 to 70 yrs with a BMI ≥30 in the past 12 months and intentional weight loss (at least 5% of their body weight) during the same period	*Weight Loss Maintenance in Adults* (WILMA): multicomponent intervention with individual motivational interviewing sessions plus professional-led peer group support sessions Community center; 12 months	Evaluation of recruitment strategies to a pragmatic RCT	Quantitative (narrative description of recruitment sources)

BMI, body mass index; NR, not reported; RCT, randomized controlled trial; UK, United Kingdom; US, United States; yr(s), year(s); YMCA, young Men's Christian association.

### 3.2 Quality appraisal

According to our assessment using the QuADS checklist, all 16 included studies had limitations in certain areas. For example, in some studies, the information on the study sample selection was completely missing or insufficiently described (e.g., only rough information on the characteristics of the study population, no information on dropouts) (15, 17, 21, 22). The data collection process was often not described at all or only in general terms in some studies (e.g., conducting interviews) (17). In addition, some studies provided no or insufficient information on the data content (e.g., missing list of interview questions) (13, 15, 19–21, 24, 26). Apart from this, some studies addressed no or only some limitations without addressing their impact on the results (12). The detailed QuADS assessment can be found in the Supplementary Material.

### 3.3 Target groups and program characteristics

#### 3.3.1 Children and adolescents

Of the eleven included studies on children and adolescents, the programs of six studies were aimed at children *and* adolescents (12, 14–16, 19, 21), while one study included children (without a more precise age limit) (13) and three studies included only adolescents (18, 20, 22). The intervention in another study was aimed at the parents of four-year-old children (17). Five studies included children and/or adolescents with overweight or obesity without further defining BMI (12, 14, 16, 18, 20). Three studies specified a BMI above the 85th percentile as an inclusion criterion (13, 21, 22). One publication (19) analyzed data from three studies with different BMI ranges. The program of one study was explicitly aimed at children and adolescents with overweight (not obesity) (15) and another at the parents of children with overweight (17). One study examined a program specifically for African-American families (18), and another included children from families with diverse ethnic backgrounds and low income (12). One study examined recruitment strategies for two interventions delivered in a medically underserved rural area (13). Two other studies defined rural residence as a further inclusion criterion (14, 19).

The programs examined in the eleven studies were carried out in different settings, including clinics, community centers and online. In all programs, the content was delivered (at least in part) in a group setting. The content of the programs covered the areas of nutrition (e.g., eating habits, healthy eating), exercise (e.g., increasing physical activity, reducing sedentary activities, exercise games for children and parents) and behavior (e.g., goal setting, self-monitoring, building positive self-esteem, parenting skills). The program duration ranged from eight weeks (22) to two years (20). Various professional groups were mentioned as carrying out the programs, most frequently dieticians (13, 14, 20–22) and psychologists (14, 19, 22).

#### 3.3.2 Adults

The five included studies (10, 23–26) on recruitment strategies and barriers in adults included different target groups: young adults aged 18 to 34 or 35 years in two studies (24, 25); older adults up to 70 (26) and 75 years (10, 23) in three studies. In two studies, only women were included (23, 24). The inclusion criteria were heterogeneous concerning BMI across the studies: ≥25 kg/m^2^ (25), ≥30 kg/m^2^ (26), 25 to 39.9 kg/m^2^ (24), 30 to 45 kg/m^2^ (10), and not specified (23). One study included adults who had lost at least 5% of their body weight in the previous 12 months (with a baseline BMI of ≥30 kg/m^2^) (26). Two studies included African-American and white women (24), and women of different ethnic backgrounds (23), respectively. Another study focused on adults living in rural areas (10).

The interventions examined in the studies of adults were carried out in different settings (e.g., online, community center, primary care practice). All programs included group meetings. In addition, content was also delivered via an interactive DVD (24), telephone contact (25) or individual counselling (26). The content of the programs comprised nutrition (e.g., personalized calorie targets, meal planning and preparation), exercise (e.g., increasing physical activity, self-monitoring) and behavior (e.g., motivational interviewing, self-monitoring, goal setting). The duration of the program ranged from ten weeks (24) to two years (10, 25). The intervention was carried out by dieticians (10, 23, 25), professionals with training in motivational interviewing (25, 26) or psychologists (23).

### 3.4 Recruitment setting and recruiting person

Most frequently mentioned settings for the recruitment of *children* and their families for multicomponent overweight or obesity programs included medical practices (pediatricians or general practitioners) and children's clinics (12–15, 17, 20, 21), followed by schools (13, 15, 18, 20, 21) and the community (18, 20). Doctors and their staff (e.g., nurses, social workers) (12, 15, 17, 21), as well as the study or program team (20, 21), were mainly responsible for recruitment. The recruitment of *adults* took place in primary care practices (10, 26), clinics (not further defined) (24), in the community (25, 26), in universities (25), and fitness centers (26). People responsible for the recruitment included, for example, primary care professionals (10) and university students and staff members trained to be culturally sensitive, speak clearly and listen respectfully (24).

### 3.5 Recruitment strategies

Fifteen of the 16 included studies reported the applied recruitment strategies, often combining several methods. One possible categorization of recruitment methods is the distinction between active and passive strategies. Active recruitment includes methods in which the recruiters actively identify and approach potential participants (e.g., by telephone, post or in person). In contrast, with passive methods, people identify themselves as potential participants after learning about a program through, e.g., the media, flyers, or posters. In summary, it can be stated that in 10 of the 16 included studies, both active and passive recruitment strategies were applied.

#### 3.5.1 Active recruitment

The most frequently applied active method for recruiting *children and adolescents* was the referral by doctors (13, 15–17, 21), e.g., by linking the recruitment with already implemented routine examinations in children (16, 17). Referral was described as the most successful strategy in one study, accounting for 47% of all enquiries and 61% of all enrolments to the program (21). In another study with a program for overweight children, referral from the pediatrician was the second most successful strategy regarding the number of enrolled participants (15). Five studies (12, 15, 17, 20, 21) also mentioned strategies to support recruitment that were targeted at healthcare professionals, e.g., by writing directly to healthcare professionals, providing material, or offering information events or training. Another strategy applied in four studies was direct contact of potential participants by letter, mail or telephone (12, 14, 16, 19). One RCT compared active and passive strategies to recruit children and their families and reached more potential participants with active methods than with passive methods such as posters, flyers, and social media (14). In addition to passive and active recruitment strategies, there is a further distinction between opt-in and opt-out methods. Opt-in means that potential participants must contact the study or program team to receive further information if they are interested in the intervention. In an opt-out strategy, potential participants must contact the team (e.g., through a pre-addressed and stamped postcard or by telephone) if they *do not* wish to receive further information. Opt-out methods were applied in two studies (14, 19) and were recommended especially for recruiting families with younger children, as they were more challenging to reach (19). Screening in schools was applied as a further active recruitment strategy in two studies (15, 21) but was the least successful strategy in one study, with less than 2% of the recruited participants (21).

For the recruitment of *adults*, direct contact (10, 23), referral by doctors (10, 26) and strategies aimed at healthcare professionals (10, 26) were mentioned by two studies each. Two studies on (young) adults achieved the greatest success by writing to potentially interested people (10, 25): 40% of the study participants were informed of the intervention by mass mailing (25) and in the second study, 66% of the potential participants that contacted the study team, reported being referred by the mailing (10). One RCT compared different types of letters to adults from ethnically diverse backgrounds and reported higher response rates with recruitment letters that were ethnically adapted. In contrast, a personalization of the letters showed no difference (23). Referral by doctors was described as the second most successful strategy in one study, with 21% of potential participants who contacted the study team being referred by a provider during a clinic visit (10). Another study recommended employing peers with similar backgrounds or people with cultural sensitivity training for the recruitment (24).

#### 3.5.2 Passive recruitment

In the studies on *children and adolescents*, the most frequently used passive recruitment strategies were articles, interviews and advertisements in print media, such as local newspapers (13, 14, 16, 18–21). Radio and television were also used for recruitment in five studies (15, 16, 18, 20, 21). Two studies described media advertising as the most successful recruitment strategy (15, 20): One study reported that most families who contacted the study team for participation stated that they had heard about the program through the media (15); however, almost 80% of these families could not be enrolled because their children were obese and not overweight (which was an inclusion criteria for the program). The second study reported enquiries, enrolments, and recruitment yields for each recruitment strategy. The media advertising accounted for 39% of enquiries, 35% of enrolments, and 12% recruitment yield, which was described as the most successful strategy. The distribution of flyers and brochures at relevant locations, such as educational institutions, health centers, or doctors' practices, was applied in more than half of the studies on children and adolescents (13–16, 19, 20). A commonly used strategy was to give presentations or provide information booths at local or school events (13, 16, 18–20). Four studies (16, 18, 20, 21) mentioned word-of-mouth and the involvement of the experiences of peers as a recruitment strategy, which was particularly relevant in a study focusing on adolescents (20). In one study that included African-American families, specific socio-cultural methods were more successful at first contact than strategies that did not consider socio-cultural background (18): 79% of the recruited families were identified through specific socio-cultural strategies. Further strategies that were used for the recruitment of children, adolescent and their families included displaying posters (e.g., in doctors' practices, community centers) (14, 16), information on websites (14, 20), school newsletters (20, 21), social media (14), press conferences (15), and the cooperation with a Public Relations department (20).

The most frequently mentioned passive strategies for the recruitment of *adults* were print media (10, 23, 25), social media (10, 25, 26), and information on websites (10, 25, 26). Interestingly, in a study on young adults, less than 1% of participants were recruited through social media. In contrast, information on websites was the second most successful strategy in the same study (22% of the recruited participants) (25). The recruitment method with the greatest yield, however, was mass mailings (40% of the recruited participants), i.e., an active strategy (see above). Further passive methods for recruiting adults that were applied included distributing flyers and brochures (25), mass mailings (25), press releases (26), word-of-mouth (23), and the offer of incentives (e.g., gift vouchers, money, tickets) (25).

One study (10) concluded that passive strategies are typically less expensive but less successful than active ones. In contrast, active strategies, such as proactive phone calls, are very time-consuming and therefore difficult to apply in clinical practice. However, the latter may be more effective in reaching specific populations, such as men, ethnic minorities, those with less education or those with higher medical risks. Due to the higher costs and effort involved with active recruiting strategies, they should be favored for target groups less likely to respond to passive strategies.

The strategies that were applied in the included studies are listed in Table 3. The table also provides additional information regarding the “success” of the methods, if available.

**Table 3:** Summary of recruitment strategies.

Recruitment strategies	Number of studies on children & adolescents (total of 11 studies)	Number of studies on adults (total of 5 studies)	Indications of effectiveness*, additional information on specific groups, …* Results from RCTs in *italics*
Passive recruitment strategies			
Print media [e.g., articles in (local) newspapers, interviews, advertisements]	7 (13, 14, 16, 18–21)	3 (10, 23, 25)	In two studies on programs for children and young people, advertising through the media was the most successful recruitment strategy (15, 20)
Radio and television (e.g., reports, interviews)	5 (15, 16, 18, 20, 21)		
Cooperation with Public Relations (PR) department	1 (20)		
Social media	1 (14)	3 (10, 25, 26)	In one study on young adults: only 0.6% of participants were recruited through social media (25)
Distributing flyers and brochures at various locations (e.g., educational institutions, doctors’ practices, health centres, youth centres, fitness centres, pharmacies)	6 (13–16, 19, 20)	1 (25)	
Mass mailings		1 (25)	
Displaying posters (e.g., in doctors’ practices, community centres)	2 (14, 16)		
Information on websites	2 (14, 20)	3 (10, 25, 26)	The second most successful strategy in one study on young adults (22% of the recruited persons) (25)
Schools newsletters	2 (20, 21)		
Presentations or information booths at local events or school events	5 (13, 16, 18–20)		
Press conference, press release	1 (15)	1 (26)	
Word of mouth, involvement of the experiences of peers	4 (16, 18, 20, 21)	1 (23)	Involvement of peers particularly relevant for adolescents (20)
Specific socio-cultural methods (e.g., cooperation with facilities/offers used by communities, socio-cultural events)	1 (18)		More successful at first contact than those strategies that did not specifically take socio-cultural background into account (18)
Offer incentives (e.g., gift vouchers, money, tickets)		1 (25)	
Active recruitment strategies			
Direct contact by letter, mail or telephone	4 (12, 14, 16, 19)	2 (10, 23)	*One RCT* (14) *compared active and passive strategies for recruiting to a program for children and their families → more potential participants were reached with the active methods (letter with opt-out option) than with the “traditional” (passive) methods (e.g., posters, flyers, advertisements on social media and websites,…)* *One RCT* (23) *compared different types of letters to adults from ethnically diverse backgrounds → greater success with ethnically adapted messages in recruitment letters; personalization of messages showed no difference (response rate of around 30,000 letters sent out by a marketing company was 0.7%)* In two studies (10, 25) on (young) adults, the greatest success was achieved by writing to potentially interested persons (66% and 44% of recruited persons, respectively)
*Opt-out strategies*	2 (14, 19)		*[see above* (14)*]* Recommended in one study (19), especially for the recruitment of families with younger children, as they were more difficult to reach
Referral by doctors	5 (13, 15–17, 21)	2 (10, 26)	E.g., by linking the recruitment with already implemented routine examinations in children (16, 17) In 2 studies on children most successful strategy (21) or second most successful strategy (15) In 1 study on adults second most successful strategy (22% of the recruited persons) (10)
Recruitment in clinics by people with culturally sensitive training using appropriate program materials and incentives		1 (24)	Training of peers for recruitment recommended; with similar (e.g., ethnic) background or cultural sensitivity training (24)
Screenings in schools	2 (15, 21)		In one study, the least successful recruitment strategy (<2% of the families recruited) (21)
Strategies to support recruitment aimed at healthcare professionals	5 (12, 15, 17, 20, 21)	2 (10, 26)	Examples: writing directly to healthcare professionals with information, providing materials, offering information events/training, financial incentives for doctors

RCT, randomized controlled trial.

### 3.6 Barriers and facilitators

The included studies reported numerous barriers to recruiting participants for multicomponent overweight and obesity group programs. These are located at different levels. At a societal level, barriers are related to the perception of overweight and obesity and the associated stigmatization that can be a barrier for the participation in a program (13, 14, 17, 22). At an institutional level, a lack of time and resources was reported (17, 26). At the level of healthcare staff, difficulties or insufficient counselling skills on the part of the healthcare professionals when discussing weight status with patients (or their parents) were mentioned (15, 17, 22, 26), as well as problems with correct identification of overweight, e.g., in younger children (15, 19). At the program level, the main potential barriers are scheduling issues, transport, and other commitments (e.g., childcare) (13, 16, 18). At the participant level, studies on programs for children and adolescents mainly reported barriers concerning the parents, e.g., lack of motivation for treatment (this was described especially for families with children who are overweight, not obese) (15, 17, 21), underestimation of children's weight status (15, 17, 21), time constraints on families, competing demands or other challenges with higher priority, or low health literacy (13, 17, 21). Barriers on the part of children and adolescents included fear of bullying and discrimination (22). For adults, potential barriers included previous weight loss failures and lack of interest in long-term lifestyle changes (24).

Some studies also reported facilitators to address these barriers, such as flexible scheduling, support with childcare and transport, proximity to program locations, incentives and continuous individual support (13, 18).

## 4 Discussion

Multicomponent lifestyle interventions are recommended for children, adolescents, and adults who are overweight or obese. However, program providers often face challenges in recruiting and motivating target groups to participate in such a program. This systematic review identified different strategies that can be used to improve the recruitment of children, adolescents, and adults with overweight or obesity to multicomponent group programs. Based on a systematic literature search, 16 studies were included and analyzed narratively. Recruitment strategies were categorized into active and passive methods. The information on how many of the people approached took part in the program (i.e., recruitment rates) was very heterogeneous, and the studies sometimes came to contradictory results, e.g., as to whether passive (e.g., via the media) or active (e.g., by referral) recruitment methods are more successful. Consequently, a combination of active and passive methods was often used or recommended in the included studies. The identified recruiting strategies did not differ significantly between children, adolescents, and adults. One reason could be that parents and caregivers need to be involved in programs for children and adolescents, and the strategies are therefore also directed at them. For socioeconomically disadvantaged groups, some targeted strategies were identified, e.g., recruitment in specific locations or through trained peers. Several possible barriers to recruitment were mentioned in the studies included, ranging from barriers on a societal level (e.g., stigmatization) to barriers at the level of healthcare professionals (e.g., lack of time and resources, insufficient counselling skills) and the participant level (e.g., organizational barriers, lack of motivation).

The interpretation of the results of this systematic review must be performed against the background of some limitations of the included literature. The assessment of the quality of the included studies using the QuADS tool showed that all studies had limitations in certain areas, e.g., insufficient information on the selection of the study sample or data collection and insufficient addressing of the limitations of the studies. Generally, the methods used for data collection and synthesis were often not described in detail. This is probably because a large proportion of the included studies—except for the two RCTs and the four qualitative studies—present the description of recruitment strategies for other studies (e.g., RCTs), rather than the use and success of strategies being investigated or compared with each other in a suitable study design. It remains unclear whether the results are transferable to program recruitment (i.e., independent of studies or evaluations) or whether different mechanisms or barriers would be relevant. In addition, the recruitment results were reported very heterogeneously in the studies and could therefore not be summarized. Furthermore, ten of the 16 studies were conducted in the US, which could influence the transferability of the results to the context of other countries. The barriers to recruitment were the focus of some qualitative studies, which were systematically surveyed and clustered in these studies (using interviews or focus groups). In the other studies, barriers were only reported narratively (sometimes as part of the discussion), and how they were collected was not described. Overall, the results on the recruitment strategies and the identified barriers should therefore not be seen as reliable, robust evidence, but primarily as a selection of different options that were supplemented with indications of which strategies could work in which areas for which target groups.

Furthermore, some identified recruiting strategies should be critically analyzed from an ethical perspective. For example, two studies on children and adolescents (14, 19) reported good recruitment rates using opt-out methods. In this case, potential participants must contact the program team (e.g., by telephone) within a specified time period if they do not wish to receive information about a study or program; otherwise, they are automatically contacted with further information. From a behavioral economics perspective, this strategy works because people generally tend to choose the option requiring no action (19). Even if the aim here is not to include people directly in a study or program unless they choose the opt-out option, but merely to contact them with information about a program, the question still arises from an ethical perspective whether this approach of providing information without being asked is justifiable.

Besides, as the fear of stigma has been mentioned as an important barrier to participation in overweight and obesity group programs in some of the included studies, the aspect of stigmatization and discrimination needs to be considered within these programs. Often, people who are overweight or obese experience stigmatization and discrimination, also within the healthcare system. Potential consequences of stigmatization in patients with obesity include, e.g., an increase in eating disorders, a decrease in physical activity, further weight gain, increased risk for depression and suicidality, and avoidance of medical consultation (27). Weight-neutral approaches focus on promoting health, improving physical and psychosocial well-being and quality of life. They aim to reduce weight-related stigma (28) and can therefore be considered to address this barrier.

To our knowledge, this is the first systematic review that summarizes recruitment strategies for both children and adults with overweight or grade one obesity to multicomponent obesity group programs. Other reviews on recruiting had other or broader inclusion criteria regarding the intervention and/or target groups (e.g., nutrition and physical activity interventions for all adults, not focusing on overweight/obesity) (29–31), or analyzed barriers and facilitators for children only (32, 33). Overall, these reviews resulted in similar conclusions to our review. For example, the systematic review of Lam et al. (30) regarding recruiting young adults to lifestyle interventions not limited to overweight/obesity programs showed that most studies used two or more strategies for recruiting participants rather than limiting to one strategy. Passive strategies were more common, including print advertising, announcements, mass mailings, and emails, which were the top three methods. Moreover, Guagliano et al. (31) suggested a multifaceted recruitment approach to provide potential participants with repeated exposure to information on the intervention. A systematic review from Clayton et al. (33) on barriers and facilitators of children's participation in nutrition, physical activity, and obesity interventions found time constraints, transportation and lack of childcare to be the most common reported barriers. The authors concluded that facilitating factors involve offering compensation or incentives, referrals by healthcare professionals, support from staff or other parents, and using bilingual staff to recruit participants in some studies (33). Personal and program logistics were also identified as an important recruiting barrier in another systematic review that analyzed barriers and facilitators to initial and continued attendance at community-based lifestyle programs. Further barriers included parental denial of their child's health problem as well as the stigma related to overweight and obesity (32). Generally, recruitment reporting and the effectiveness of different methods should be improved (29, 31).

Despite the unique feature of this systematic review, the results of the review should be viewed in the context of its limitations. Firstly, the review focused on specific interventions, namely, multicomponent overweight and obesity group programs, covering nutrition, physical activity, and behavioral therapy elements. For this reason, only studies reporting on recruitment strategies focusing on these programs were included. Studies on programs in other settings (e.g., individual, inpatient, workplace, school) were omitted, although these studies could also provide valuable information regarding the recruitment of target groups. In addition, behavioral change interventions remain valuable for individuals with higher grades of obesity, particularly as adjunctive treatment and for weight maintenance following more intensive interventions. This represents an important area for future research beyond our current review's scope. Secondly, despite our initial intention to focus on recruitment strategies targeting socioeconomically disadvantaged populations, as stated in the introduction, our systematic review yielded insufficient data specifically addressing this demographic. This represents an important gap in the current literature and highlights the need for future research to explicitly examine effective recruitment strategies for socioeconomically disadvantaged individuals in multicomponent group programs for overweight and obesity. Thirdly, the limited reporting of facilitators compared to barriers in the reviewed literature represents a significant research gap. In addition, quantitative comparison of recruitment success across strategies was hindered by inconsistent reporting in primary studies, with varied definitions of recruitment outcomes and limited information on eligible populations. Moreover, another limitation is the lack of comparative effectiveness data in the included studies, which prevented us from determining which recruitment strategies work best for specific populations or contexts. Future research should evaluate the comparative effectiveness of different recruitment approaches to provide evidence-based guidance for practitioners. Finally, we used the QuADS tool to assess the methodological quality of the included studies because we aimed to consider various study designs (quantitative, qualitative, mixed-methods) and preferred to use the same tool for all studies. However, the QUADS tool is not designed to determine a final score or categorization, making it more challenging to account for methodological limitations. In the summary table, no distinction was made regarding the type of study from which the information was extracted, except for the two RCTs.

## 5 Conclusion

This systematic review summarizes methods and strategies for recruiting children, adolescents, and adults with overweight and obesity into multicomponent group programs. It is generally recommended to combine passive and active strategies, e.g., referral by health professionals and advertisements in the media, to reach the respective target groups. Due to some limitations of the included literature, the results of this systematic review are not robust evidence with “guaranteed success” but should be understood as an overview of valuable options that can be applied and considered by program providers when designing or adapting programs, depending on the framework conditions and target groups.

## Data Availability

The original contributions presented in the study are included in the article/Supplementary Material, further inquiries can be directed to the corresponding author.

## Supplementary material

The Supplementary Material for this article can be found at: https://www.frontiersin.org/articles/10.3389/frhs.2025.1404181/full#supplementary-material

Supplementary Data Sheet 1 Search strategy and quality assessment.
