# Supplementary material for: Strategies to improve recruitment to multicomponent group programs for overweight and obesity: a systematic review
Source: Front Health Serv. 2025 May 29;5:1404181. doi: 10.3389/frhs.2025.1404181 (PMC12159834; doi:10.3389/frhs.2025.1404181)
Supplement: Supplementary Data Sheet 1 — Search strategy and quality assessment. [file Datasheet1.pdf]

Supplementary Material

SUPPLEMENTARY TABLE 1 Search strategy MEDLINE

Database: Ovid MEDLINE(R) ALL <1946 to May 24, 2023>		
Search date: 25/05/2023		
ID	Search	Results
1	((obes* or overweight or over-weight or weight).mp. or exp Obesity/pc or exp Overweight/pc) adj3 (program* or interventi* or trial*).mp.	46,826
2	exp Weight Reduction Programs/	3,057
3	1 or 2	46,983
4	*Patient Selection/	18,741
5	target group*.mp.	7,474
6	((recruit or recruiting or recruitment* or participation*) adj3 (strateg* or method* or rate*)).mp.	21,000
7	4 or 5 or 6	45,540
8	3 and 7	461
9	limit 8 to (english or german)	457
10	remove duplicates from 9	456

SUPPLE MENTARY TABLE 2 Search strategy CINAHL

Database: PsycInfo		
Search date: 26/05/2023		
Limiters/Expanders:<br>Expanders - Apply equivalent subjects<br>Search modes - Boolean/Phrase		
#	Query	Results
S1	MM Obesity	
S2	obes*	159,381
S3	Overweight	80,849
S4	TX over-weight	157
S5	S1 OR S2 OR S3 OR S4	163,528
S6	program* OR interventi* OR trial*	1,483,963
S7	S5 AND S6	44,411
S8	MH Weight Reduction Programs	3,315
S9	S7 OR S8	45,389
S10	MM Patient Selection	5,818
S11	"target group*"	3,036
S12	(recruit OR recruiting OR recruitment* OR participation*) N3 (strateg* OR method* OR rate*)	14,248
S13	S10 OR S11 OR S12	22,583
S14	S9 AND S13	470

S15	S9 AND S13<br>Narrow by Language: - german<br>Narrow by Language: - english	468

SUPPLEMENTARY TABLE 3 Search strategy The Cochrane Library

Database: The Cochrane Library	
Search date: 25/05/2023	
ID	Search
1	((obes* OR overweight OR over-weight OR weight) NEAR (program* OR interventi* OR trial*)):ti,ab,kw
2	MeSH descriptor: [Weight Reduction Programs] explode all trees
3	#1 OR #2
4	MeSH descriptor: [Patient Selection] this term only
5	("target group*"):ti,ab,kw (Word variations have been searched)
6	((recruit OR recruiting OR recruitment* OR participation*) NEAR (strateg* OR method* OR rate*)):ti,ab,kw
7	#4 OR #5 OR #6
8	#3 AND #7
9	(conference proceeding):pt
10	(abstract):so
11	(clinicaltrials OR trialsearch OR ANZCTR OR ensaiosclinicos OR Actrn OR chictr OR cris OR ctri OR registroclinico OR clinicaltrialsregister OR DRKS OR IRCT OR Isrctn OR rctportal OR JapicCTI OR JMACCT OR jRCT OR JPRN OR Nct OR UMIN OR trialregister OR PACTR OR R.B.R.OR REPEC OR SLCTR OR Tcr):so
12	#9 OR #10 OR #11
13	#8 NOT #12
	358 Hits

SUPPLEMENTARY TABLE 4 Search strategy PsycInfo

Database: PsycInfo		
Search date: 26/05/2023		
Limiters/Expanders:<br>Expanders - Apply equivalent subjects<br>Search modes - Boolean/Phrase		
#	Query	Results
S1	(obes* OR overweight OR over-weight OR weight) N3 (program* OR interventi* OR trial*)	9,045
S2	MA Weight Reduction Programs	504
S3	S1 OR S2	9,045
S4	MA Patient Selection	3,937
S5	"target group*"	3,561
S6	(recruit OR recruiting OR recruitment* OR participation*) N3 (strateg* OR method* OR rate*)	10,395
S7	S4 OR S5 OR S6	17,327
S8	S3 AND S7	130

SUPPLEMENTARY TABLE 5 Search strategy Web of Science

Database: Web of Science		
Search date: 26/05/2023		
#	Search	Results
1	TS=((obes* OR overweight OR over-weight OR weight) NEAR/3 (program* OR interventi* OR trial*))	35,650
2	(recruit OR recruiting OR recruitment* OR participation*) NEAR/3 (strateg* OR method* OR rate*) (Topic)	55,546
3	"target group*" (Topic)	14,126
4	#2 OR #3	69,502
5	#1 AND #4	462
	#1 AND #4 and Meeting Abstract (Exclude – Document Types)	456

SUPPLEMENTARY TABLE 6 Quality assessment of the included studies on the recruitment of children and adolescents (QuADS Criteria)

QuADS Criteria	Rice 2008	Finne 2009	Nguyen 2012	Gerards 2012	Smith 2014	Fleming 2015	Huffman 2016	Barlow 2017	McCullough 2017	Brock 2021	Darden 2022
1. Theoretical or conceptual underpinning to the research	0	0	0	2[1]	2[2]	0	0	0	2[3]	0	0
2. Statement of research aim/s	3	1	3	3	2	3	3	3	3	3	3
3. Clear description of research setting and target population	2[4]	3	3	3	3	3	2	3	3	3	3
4. The study design is appropriate to address the stated research aim/s	2[5]	2[5]	2[5]	3	3	3	2[6]	3	2[6]	3	3
5. Appropriate sampling to address the research aim/s	0	1	2	1	1	3	2	1	2	2	1
6. Rationale for choice of data collection tool/s	0	0	0	3[7]	0	0	0	0	0	2[8]	0
7. The format and content of data collection tool is appropriate to address the stated research aim/s	2	2[9]	2[9]	3	3	3	3	3	2[9]	2[9]	3
8. Description of data collection procedure	1	1	2	3	3	3	3	3	3	3	3
9. Recruitment data provided	3	3	2	1	1	3	2	2	3	0	2
10. Justification for analytic method selected	0	0	0	0	0	0	0	0	0	0	0
11. The method of analysis was appropriate to answer the research aim/s	2[10]	2[10]	2[10]	3	3	3	3	3	3	3	3

[1] The research model for the study was based on an implementation theory developed by Fleuren et al.

[2] The theoretical foundation for this study was based on the Ecological System Theory (EST) proposed by Brofenbrenner.

[3] Description of Opt-in and Opt-Out recruitment methodology.

[4] Lacking detailed description of the target population.

[5] More suitable alternative could be to compare different recruitment strategies.

[6] Second analysis for recruitment data available for a RCT.

[7] A dissemination strategy was developed in order to optimally communicate the procedure with the YHC professionals.

[8] Choice of data collection tool (script for interviews) was based on a preliminary community-based participatory research.

[9] No detailed information about the content.

[10] Evaluation of recruitment strategies.

QuADS Criteria	Rice 2008	Finne 2009	Nguyen 2012	Gerards 2012	Smith 2014	Fleming 2015	Huffman 2016	Barlow 2017	McCullough 2017	Brock 2021	Darden 2022
12. Evidence that the research stakeholders have been considered in research design or conduct	2	2	2	3[11]	3[12]	1	0	3[13]	3[14]	3[15]	0
13. Strengths and limitations critically discussed	0	2	2	2	2	0	2	1	2	3	3

0 *= no information about the QuADS criteria reported in the study vs* ***3*** *= most detailed information about the QuADS criteria described in the study;*
Detailed description of the QuADS criteria see: Quality Assessment with Diverse Studies (QuADS) Criteria. 2021 [cited 20/03/2024]. Available from: https://static-content.springer.com/esm/art%3A10.1186%2Fs12913-021-06122-y/MediaObjects/12913_2021_6122_MOESM2_ESM.pdf.

[11] All YHC professionals working in the 14 child health clinics were eligible to participate in the interviews.

[12] A total of 26 interviews were conducted with 39 health professionals, local service providers and researcher stakeholders.

[13] The research staff met with providers and their staff members at each primary care practice to discuss the proposed study and to elicit concerns as the study protocol was developed and finalized.

[14] Institutional review board.

[15] Advisory group.

TABLE 7 Quality assessment of the included studies on the recruitment of adults (QuADS Criteria)

QuADS Criteria	Chang 2009	Brown 2012	Corsino 2013	Randell 2015	Befort 2020
1. Theoretical or conceptual underpinning to the research	0	1[16]	0	0	0
2. Statement of research aim/s	3	3	3	3	3
3. Clear description of research setting and target population	3	3	3	3	3
4. The study design is appropriate to address the stated research aim/s	2[17]	3	2[17]	2[18]	3
5. Appropriate sampling to address the research aim/s	1	3	2	3	2
6. Rationale for choice of data collection tool/s	0	3	3	0	3[19]
7. The format and content of data collection tool is appropriate to address the stated research aim/s	0[20]	3	3	0	3
8. Description of data collection procedure	1	0	3	0	2
9. Recruitment data provided	3	3	0[21]	3	3
10. Justification for analytic method selected	0	0	0	0	0
11. The method of analysis was appropriate to answer the research aim/s	3	3	3	2[22]	3
12. Evidence that the research stakeholders have been considered in research design or conduct	0	1	0	3[23]	1
13. Strengths and limitations critically discussed	1	2	0[24]	0[24]	2

***0** = no information about the QuADS criteria reported in the study vs **3** = most detailed information about the QuADS criteria described in the study;*
Detailed description of the QuADS criteria see: Quality Assessment with Diverse Studies (QuADS) Criteria. 2021 [cited 20/03/2024]. Available from: https://static-content.springer.com/esm/art%3A10.1186%2Fs12913-021-06122-y/MediaObjects/12913_2021_6122_MOESM2_ESM.pdf.

[16] Several recruiting strategies are described in the introduction.

[17] More suitable alternative could be to compare different recruitment strategies.

[18] A reflection of the challenges experiences during the delivery of a trial, including the recruiting process.

[19] Study protocol available.

[20] No further information about the content of the telephone interviews.

[21] Not applicable, because in this study young adults were recruited to inform a recruitment protocol for an RCT.

[22] Many of the recruitment strategies devised in the plan were implemented at the same time. Therefore, it was not possible to gather the appropriate data in order to make a detailed analysis of the impact of each aspect of the recruitment plan. Impact has been described here based on the experience of the study team.

[23] All trial processes and documentation were approved by Wales Research Ethics Committee 3 and the relevant research and development committees within the NHS. In Wales, the Welsh Government body, National institute for social care and health research (NISCHR), provided a nationwide centralized process for obtaining research governance, NHS costs and research network support. In England, these were dealt with by different agencies at a local level.

[24] No limitations mentioned.
